# Supplementary material for: Occupational gender segregation and economic growth in U.S. local labor markets, 1980 through 2010
Source: PLoS One. 2020 Jan 14;15(1):e0227615. doi: 10.1371/journal.pone.0227615 (PMC6959984; doi:10.1371/journal.pone.0227615)
Supplement: S1 Appendix — (DOCX) [file pone.0227615.s001.docx]

I adopted the procedures outlined in Dorn [57] and used in previous research [21,54] to assign 1980, 1990, 2000, and 2010 PUMS respondents to labor markets. The most precise geocode for respondents’ place of residence in the PUMS data is the Public Use Microdata Area (PUMA) for years 1990, 2000, and 2010, and the county group for year 1980. For each respondent, I calculated the probability that they resided in a labor market based on the location of their associated PUMA/county group. For respondents in PUMAs/county groups located entirely within labor markets, their associated probability was 1. For those in PUMAs/county groups straddling labor market boundaries, their probability of labor market assignment was determined by the proportion of PUMA/county group residents residing in each labor market, expressed as:

$$a_{jk}=\sum_{c=1}^{C} \frac{r_{jc}}{r_{j}}\frac{r_{ck}}{r_{c}}$$

Moving backwards in the equation, $\frac{r_{ck}}{r_{c}}$ represents the share of county *c*’s population ($r_{c}$), residing in labor market *k* ($r_{ck}$). Because each county matches directly to a single labor market, this will either be zero or one. Next, $\frac{r_{jc}}{r_{j}}$ represents the share of PUMA/county group *j* residents within county *c*. To determine this figure, I used the Geocorr application from the Missouri Census Data Center (see <http://mcdc.missouri.edu/applications/geocorr.html>) that calculated $\frac{r_{jc}}{r_{j}}$ using detailed census-block level population estimates for each year included in the data. Summing $\frac{r_{jc}}{r_{j}}$ for all counties associated with a particular PUMA/county group generates the probability that respondents in PUMA/county group *j* reside in labor market *k* ($a_{jk}$). Respondents in PUMAs/county groups straddling labor markets will have a $a_{jk}$ between zero and one for more than one labor market. These respondents are duplicated in the dataset and weighted by $a_{jk}$.

This approach allows for full information to be retained when generating labor market estimates while accounting for the fact that precise geo-codes for respondents are kept confidential by the Census Bureau. In most cases, PUMAs/county groups fall entirely within labor markets. In the PUMS data used in this study, only 15 percent of respondents resided in a PUMA that straddled labor markets.
