# Supplementary material for: Occupational gender segregation and economic growth in U.S. local labor markets, 1980 through 2010
Source: PLoS One. 2020 Jan 14;15(1):e0227615. doi: 10.1371/journal.pone.0227615 (PMC6959984; doi:10.1371/journal.pone.0227615)
Supplement: S2 Appendix — (DOCX) [file pone.0227615.s002.docx]

To test whether findings on the relationship of occupational gender segregation to wages persists at other levels of the wage distribution, I repeated the models reported in Table 3, models 3 through 8, of the main text using wages at the 25^th^ and 75^th^ percentiles as the dependent variable. Results testing the direct effects of occupational gender segregation on wages are reported in Table B1, while those testing the moderating effects of occupational gender segregation on the relationship of industry expansion to wages are reported in Table B2. In general, the pattern of occupational gender segregation hindering industry wage growth was consistent across the wage distribution. The strongest effects were observed for wages in the 75^th^ percentile. This is consistent with the hypothesized theoretical mechanism, that occupational gender segregation affects the degree to which information is shared in high-skilled work settings, because those in the higher end of the wage distribution are more likely to be in high-skilled positions where knowledge and information sharing play a larger role.

**S2 Table 1**. Fixed Effects Models Testing Direct Effects of Occupational Gender Segregation on Hourly Wages at 25th, 50th, and 75th Percentiles, 1980-2010

|  | | | | | | | | | | | |  |
| --- | --- | --- | --- | --- | --- | --- | --- | --- | --- | --- | --- | --- |
|  | FIRE | | |  | Tech | | |  | Retail Trade | | | |
|  | Wages, 25th Percentile | Wages, Median | Wages, 75th Percentile |  | Wages, 25th Percentile | Wages, Median | Wages, 75th Percentile |  | Wages, 25th Percentile | Wages, Median | Wages, 75th Percentile | |
|  | Model 1 | Model 2 | Model 3 |  | Model 4 | Model 5 | Model 6 |  | Model 7 | Model 8 | Model 9 | |
| Industry Characteristics |  |  |  |  |  |  |  |  |  |  |  | |
| Occupational Gender Segregation | -1.262* | -2.441** | -2.889† |  | 0.131 | 0.360 | 0.255 |  | 1.592** | 1.439 | 2.044 | |
|  | (0.563) | (0.787) | (1.640) |  | (1.092) | (1.538) | (1.798) |  | (0.611) | (0.915) | (1.479) | |
| % Employed in Industry | 0.253*** | 0.165* | 0.137 |  | 0.276*** | 0.372** | 0.478*** |  | -0.001 | -0.027 | 0.024 | |
|  | (0.050) | (0.078) | (0.146) |  | (0.067) | (0.119) | (0.136) |  | (0.026) | (0.044) | (0.081) | |
| Occupational Racial Segregation | -0.276** | -0.223 | -0.574 |  | 0.034 | -0.042 | 0.332 |  | 0.022 | -0.161 | -0.231 | |
|  | (0.090) | (0.147) | (0.306) |  | (0.202) | (0.284) | (0.383) |  | (0.198) | (0.298) | (0.428) | |
| % College Educated | 5.128*** | 7.230*** | 13.659*** |  | 5.536** | 8.125** | 12.565*** |  | 2.848† | 6.556* | 14.324** | |
|  | (1.081) | (1.778) | (3.088) |  | (1.894) | (2.584) | (3.345) |  | (1.663) | (3.080) | (5.423) | |
| % Women | -1.839* | -4.973*** | -14.718*** |  | -7.563*** | -12.862*** | -11.517*** |  | -1.864* | -3.886* | -5.590* | |
|  | (0.888) | (1.270) | (2.730) |  | (1.421) | (2.347) | (2.554) |  | (0.910) | (1.535) | (2.577) | |
| % White | 0.817 | -0.231 | -1.215 |  | 1.836 | 5.423* | 4.510 |  | 0.976 | 2.286 | 3.031 | |
|  | (1.117) | (1.604) | (2.723) |  | (1.594) | (2.567) | (3.577) |  | (0.977) | (1.854) | (2.994) | |
| Labor Market Characteristics | | | |  |  |  |  |  |  |  |  | |
| Population (logged) | 0.223 | 0.011 | -0.346 |  | 0.274 | -0.878 | -0.668 |  | 0.934*** | 1.031* | 0.942 | |
|  | (0.352) | (0.558) | (1.150) |  | (0.576) | (0.857) | (1.280) |  | (0.244) | (0.410) | (0.681) | |
| % Foreign Born | 3.428 | 14.939* | 41.381*** |  | 10.800 | 26.389** | 40.459** |  | -2.917 | -3.925 | -4.667 | |
|  | (3.831) | (5.902) | (12.064) |  | (6.237) | (9.218) | (13.040) |  | (2.247) | (3.835) | (6.412) | |
| % Aged 25-40 | 1.058 | 2.782 | -0.728 |  | 5.610 | 8.560 | 1.642 |  | 9.038** | 19.322*** | 37.776*** | |
|  | (3.369) | (6.728) | (11.050) |  | (5.278) | (9.119) | (16.737) |  | (2.723) | (5.765) | (9.224) | |
| % White | -2.461 | -1.232 | 7.754 |  | 1.405 | 1.357 | 4.055 |  | 0.607 | 1.341 | 1.955 | |
|  | (2.058) | (2.935) | (5.370) |  | (2.967) | (4.252) | (6.041) |  | (1.010) | (1.752) | (3.022) | |
| Unemployment Rate | -9.222*** | -12.152*** | -19.606** |  | 2.284 | 3.756 | -0.485 |  | -0.669 | -4.449 | -9.608* | |
|  | (1.930) | (3.197) | (6.271) |  | (3.763) | (5.102) | (7.131) |  | (1.448) | (2.401) | (3.946) | |
| % College Educated | 19.922*** | 32.267*** | 44.987*** |  | 22.531*** | 30.351*** | 33.898*** |  | 6.507*** | 11.810*** | 17.792*** | |
|  | (2.863) | (4.519) | (7.055) |  | (4.328) | (6.594) | (8.849) |  | (1.775) | (3.174) | (4.965) | |
| % Employed in Export-Intensive Industries | -1.332 | -6.829** | -10.467* |  | 2.231 | 3.694 | -2.490 |  | 1.269 | 1.621 | 4.692 | |
|  | (1.497) | (2.216) | (4.064) |  | (2.725) | (4.374) | (5.104) |  | (1.098) | (1.662) | (2.894) | |
| Women's Labor Force Participation | -5.343*** | -7.089** | -17.259*** |  | -5.988* | -15.267*** | -10.232† |  | 1.539* | -1.597 | -6.899** | |
|  | (1.405) | (2.119) | (4.009) |  | (2.485) | (3.978) | (5.332) |  | (0.751) | (1.246) | (2.255) | |
| Service to Goods Employment Ratio | -0.052 | -0.031 | -0.035 |  | -0.222 | -0.018 | 0.050 |  | -0.167** | -0.151 | -0.167 | |
|  | (0.101) | (0.147) | (0.305) |  | (0.199) | (0.308) | (0.381) |  | (0.062) | (0.108) | (0.171) | |
| Constant | 9.820* | 18.897** | 35.723* |  | 6.185 | 28.69* | 30.63* |  | -8.960** | -8.382 | -5.400 | |
|  | (4.873) | (6.986) | (14.107) |  | (8.076) | (11.38) | (14.50) |  | (2.811) | (4.578) | (7.798) | |
| Fixed Effects | Year Labor Market | Year Labor Market | Year Labor Market |  | Year Labor Market | Year Labor Market | Year Labor Market |  | Year Labor Market | Year Labor Market | Year Labor Market | |
| N | 716 | 716 | 716 |  | 716 | 716 | 716 |  | 716 | 716 | 716 | |
| R-sq | 0.745 | 0.811 | 0.740 |  | 0.546 | 0.533 | 0.590 |  | 0.856 | 0.761 | 0.726 | |

*NOTE*: Percent employed in sector multiplied by 100. Standard errors clustered by labor market, reported in parentheses.

†p<.1, *p<0.05, **p<0.01, ***p<0.001

**S2 Table 2.** Fixed Effects Models Testing Moderating Effects of Occupational Gender Segregation on the relationship of Industry Expansion to Hourly Wages at 25th, 50th, and 75th Percentiles, 1980-2010

|  | FIRE | | |  | Tech | | |  | Retail Trade | | |
| --- | --- | --- | --- | --- | --- | --- | --- | --- | --- | --- | --- |
|  | Wages, 25th Percentile | Wages, Median | Wages, 75th Percentile |  | Wages, 25th Percentile | Wages, Median | Wages, 75th Percentile |  | Wages, 25th Percentile | Wages, Median | Wages, 75th Percentile |
|  | Model 1 | Model 2 | Model 3 |  | Model 4 | Model 5 | Model 6 |  | Model 7 | Model 8 | Model 9 |
| Industry Characteristics |  |  |  |  |  |  |  |  |  |  |  |
| Occupational Gender Segregation | 4.968*** | 5.185** | 5.861 |  | 2.510 | 7.440** | 11.713*** |  | -3.223 | -5.072 | -4.690 |
|  | (1.154) | (1.765) | (3.595) |  | (1.678) | (2.373) | (3.053) |  | (4.451) | (7.705) | (13.251) |
| % Employed in Industry | 0.854*** | 0.900*** | 0.981** |  | 0.594** | 1.320*** | 2.012*** |  | -0.127 | -0.197 | -0.153 |
|  | (0.107) | (0.155) | (0.326) |  | (0.211) | (0.258) | (0.316) |  | (0.117) | (0.206) | (0.360) |
| Gender Segregation * % Employed in Industry | -1.274*** | -1.560*** | -1.790** |  | -0.551 | -1.640*** | -2.654*** |  | 0.291 | 0.394 | 0.407 |
|  | (0.219) | (0.325) | (0.641) |  | (0.347) | (0.404) | (0.551) |  | (0.264) | (0.456) | (0.794) |
| Occupational Racial Segregation | -0.189* | -0.116 | -0.452 |  | 0.053 | 0.014 | 0.423 |  | 0.034 | -0.144 | -0.214 |
|  | (0.092) | (0.155) | (0.317) |  | (0.204) | (0.284) | (0.350) |  | (0.195) | (0.291) | (0.417) |
| % College Educated | 4.482*** | 6.438*** | 12.751*** |  | 5.582** | 8.263** | 12.788*** |  | 2.949† | 6.692* | 14.465** |
|  | (1.073) | (1.777) | (3.088) |  | (1.919) | (2.535) | (3.266) |  | (1.719) | (3.158) | (5.476) |
| % Women | -0.699 | -3.578** | -13.117*** |  | -7.355*** | -12.246*** | -10.518*** |  | -1.989* | -4.055* | -5.764* |
|  | (0.907) | (1.310) | (2.865) |  | (1.412) | (2.327) | (2.497) |  | (0.924) | (1.589) | (2.642) |
| % White | 1.591 | 0.716 | -0.128 |  | 1.850 | 5.464* | 4.576 |  | 1.028 | 2.355 | 3.103 |
|  | (1.002) | (1.518) | (2.702) |  | (1.571) | (2.467) | (3.310) |  | (0.981) | (1.846) | (2.976) |
| Labor Market Characteristics | | |  |  |  |  |  |  |  |  |  |
| Population (logged) | -0.029 | -0.297 | -0.700 |  | 0.344 | -0.669 | -0.330 |  | 1.070*** | 1.215* | 1.133 |
|  | (0.334) | (0.552) | (1.148) |  | (0.559) | (0.807) | (1.189) |  | (0.316) | (0.549) | (0.892) |
| % Foreign Born | 3.424 | 14.933** | 41.374*** |  | 8.538 | 19.657* | 29.565* |  | -3.135 | -4.220 | -4.972 |
|  | (3.297) | (5.435) | (11.540) |  | (5.785) | (8.470) | (11.866) |  | (2.352) | (4.041) | (6.675) |
| % Aged 25-40 | -0.032 | 1.447 | -2.259 |  | 6.334 | 10.715 | 5.130 |  | 9.235*** | 19.589*** | 38.052*** |
|  | (3.206) | (6.608) | (10.942) |  | (5.333) | (9.155) | (16.588) |  | (2.726) | (5.696) | (9.048) |
| % White | -1.984 | -0.648 | 8.424† |  | 1.675 | 2.158 | 5.352 |  | 0.587 | 1.314 | 1.926 |
|  | (1.701) | (2.535) | (5.000) |  | (2.918) | (3.892) | (5.361) |  | (1.015) | (1.766) | (3.036) |
| Unemployment Rate | -9.917*** | -13.002*** | -20.582** |  | 3.264 | 6.675 | 4.239 |  | -0.536 | -4.269 | -9.422* |
|  | (1.821) | (3.043) | (6.232) |  | (3.748) | (5.006) | (6.739) |  | (1.457) | (2.405) | (3.937) |
| % College Educated | 16.504*** | 28.083*** | 40.186*** |  | 20.682*** | 24.848*** | 24.992** |  | 5.879** | 10.960** | 16.914** |
|  | (2.711) | (4.477) | (7.293) |  | (4.619) | (6.986) | (8.506) |  | (1.916) | (3.425) | (5.297) |
| % Employed in Export-Intensive Industries | -1.952 | -7.588*** | -11.338** |  | 1.467 | 1.418 | -6.173 |  | 1.485 | 1.914 | 4.995 |
|  | (1.360) | (2.045) | (3.907) |  | (2.632) | (4.125) | (4.632) |  | (1.082) | (1.631) | (2.852) |
| Women's Labor Force Participation | -5.902*** | -7.774*** | -18.044*** |  | -5.314* | -13.262*** | -6.987 |  | 1.950* | -1.041 | -6.324* |
|  | (1.298) | (1.981) | (3.925) |  | (2.525) | (3.894) | (5.132) |  | (0.911) | (1.464) | (2.553) |
| Service to Goods Employment Ratio | -0.081 | -0.066 | -0.075 |  | -0.289 | -0.217 | -0.272 |  | -0.139* | -0.112 | -0.127 |
|  | (0.096) | (0.142) | (0.298) |  | (0.194) | (0.294) | (0.337) |  | (0.061) | (0.103) | (0.169) |
| Constant | 9.845* | 18.928** | 35.759* |  | 3.549 | 20.850 | 17.934 |  | -8.951** | -8.369 | -5.387 |
|  | (4.381) | (6.783) | (13.953) |  | (7.989) | (10.954) | (13.147) |  | (2.885) | (4.728) | (7.989) |
| Fixed Effects | Year Labor Market | Year Labor Market | Year Labor Market |  | Year Labor Market | Year Labor Market | Year Labor Market |  | Year Labor Market | Year Labor Market | Year Labor Market |
| N | 716 | 716 | 716 |  | 716 | 716 | 716 |  | 716 | 716 | 716 |
| R-sq | 0.765 | 0.821 | 0.744 |  | 0.550 | 0.549 | 0.612 |  | 0.856 | 0.762 | 0.727 |

*NOTE*: Percent employed in sector multiplied by 100. Standard errors clustered by labor market, reported in parentheses.

†p<.1, *p<0.05, **p<0.01, ***p<0.001
