# Supplementary material for: Occupational gender segregation and economic growth in U.S. local labor markets, 1980 through 2010
Source: PLoS One. 2020 Jan 14;15(1):e0227615. doi: 10.1371/journal.pone.0227615 (PMC6959984; doi:10.1371/journal.pone.0227615)
Supplement: Attachment — Submitted filename: PONE-D-19-24965_cover letter to editor.pdf [file pone.0227615.s003.pdf]

Xi Yang
Department of Economics
University of North Texas
Wooten Hall 347
Denton, Texas, 76203
Email: xi.yang@unt.edu

September 30, 2019

Dear Dr. Shihe Fu,

Thank you for the opportunity to review the paper for the PLOS ONE. Attached please see the referee report for the paper "Gender segregation of managerial professional occupations and economic growth in U.S. labor markets, 1980 through 2010" (PONE-D-19-24965).

This paper studies the impact of gender segregation on economic growth in the U.S. In contrast to previous studies that mostly using organization-level data (e.g., firm-level), this paper conducts empirical analysis at the local labor market level, which is defined as labor sheds. It attempts to measure managerial/professional occupational gender segregation at the local labor market level and provides some empirical work to relate the gender segregation with some local labor market outcomes.

Considering the importance of idea-exchanging in promoting local economic growth, the research question in this paper is interesting and has the potential to add value to the literature if well done. Unfortunately, I do not think the paper has adopted an appropriate variable to measure gender segregation at the local labor market level. More importantly, the empirical analysis in this paper is far from persuasive without any discussion on the potential endogeneity problem. Thus, I recommend this paper to be rejected.

Thank you again for this opportunity!

Best wishes,

Xi Yang
