# Supplementary material for: Occupational gender segregation and economic growth in U.S. local labor markets, 1980 through 2010
Source: PLoS One. 2020 Jan 14;15(1):e0227615. doi: 10.1371/journal.pone.0227615 (PMC6959984; doi:10.1371/journal.pone.0227615)
Supplement: Attachment — Submitted filename: PONE-D-19-24965_referee report.pdf [file pone.0227615.s004.pdf]

**Referee Report for PONE-D-19-24965 Gender segregation of managerial/professional occupations and economic growth in U.S. labor markets, 1980 through 2010" (William Scarborough, 2019)**

## Summary

This paper studies the impact of gender segregation on economic growth in the U.S. In contrast to previous studies that mostly using organization-level data (e.g., firm-level), this paper conducts empirical analysis at the local labor market level, which is defined as labor sheds. It attempt to measure managerial/professional occupational gender segregation at the local labor market level and provides two major empirical results. First, a higher level of segregation is associated with reduction in the employment in finance and technological service sectors. Second, a higher level of segregation is associated with worse economic well-being measured as labor market median wages, household income, and business growth.

Considering the importance of idea-exchanging in promoting local economic growth [Davis and Dingel, 2019], the research question in this paper is interesting and has the potential to add value to the literature if well done. However, in its current form, it is not clear to me that the paper has adopted an appropriate measurement for gender segregation, and its empirical analysis also needs substantial improvements.

## Main Comments

1. The occupational gender segregation index adopted in the paper does not seem to fully capture the gender segregation problem at the local labor market level. The index of dissimilarity used in this paper captures whether male and female workers are equally distributed among different managerial /professional occupations, but it fails to consider the overall representativeness of female workers in the managerial /professional occupations within the local labor market. To capture the overall representativeness of female workers among managerial /professional occupations within the local labor market, it is better to use the index of dissimilarity among all occupations instead of just managerial /professional occupations.[1]

2. Nevertheless, I think the gender segregation index deserves more description since it is the main independent variable in the paper. In particular, it would be interesting to know the index is calculated, what is its spatial distribution across the nation, and what are the potential explanations for these spatial patterns. The author could provide a map with

[1]See Taylor et al. [2019] for a better explanation of this index.

different colors indicating different levels of segregation or simply list the top 10 and bottom 10 labor markets when ranking the gender segregation index.

3. I'm a little surprised to see that between 1980 to 2010, the employment share in FIRE has only increased by 0.58 on average as reported in table 1. A report provided by the bureau of labor statistics shows that the employment share in the financial industry has increased by 7 percent between 1979 to 2001.[2]

4. The empirical work in this paper faces the endogeneity problem caused by reserve causality. That is, it is possible that the rising of the high skilled service sectors (finance and technological industries) promoted gender integration in the workplace, not the other way around as claimed in this paper. In addition, time-varying omitted variables can cause the endogeneity problem. For example, it is possible that certain demographic changes (the rising proportion of college-educated workers) are promoting gender integration and economic growth at the same time. If this is the case, the effect of gender segregation will be overestimated in the paper.

   I understand it might be challenging to come up with valid instruments variables to solve the above endogeneity issues. But I think the author should at least discuss the problem and acknowledge the limitation of current empirical methods.

5. The main estimation model lacks important control variables that are commonly included in studies of the local labor market, such as the share of college workers and the share of foreign-born workers.

6. Previous studies have identified that international trade and automation are two important driving forces behind the rising of high-skilled service sectors [Acemoglu and Autor, 2011, Goos et al., 2014]. The author might want to incorporate some variables to control for effects of these explanations in the main regression model or provide discussions on these alternative explanations.

7. The author has not left me with a clear picture of why employment expansion of finance and technological service sectors is the most proper dependent variables to capture the labor market effect of gender segregation. First, I understand that gender diversity can foster exchanging of ideas among diversified groups, and thus it may have positive impacts on high-skilled sectors (finance and technological service sectors) where knowledge sharing are particularly important. However, I do not think it will necessarily increase employment in the high-skill service sectors, instead, it could increase the productivity of workers in those sectors. If this is the case, it's better to use hourly wage in high-skill sectors as

[2]See Current Employment Statistics survey: 100 years of employment, hours, and earnings.

dependent variables instead of using employment share. The author looks wages at the local labor market level and interprets it as an indirect impact of gender integration. I don't think this is appropriate, as gender integration can have a direct impact on wages.

8. It would be interesting to see whether the labor market impacts of gender segregation are smaller or insignificant for other sectors where knowledge sharing are less important.

## Minor Comments

1. A labor market can be local or national. The author might want to specify that this paper focuses on the local labor markets.

2. In terms of the unit of analysis, studies in urban economics commonly use the metropolitan statistical area or the commuting zone. The author might want to discuss the differences between labor sheds and those commonly used units or conduct some robustness checks using alternative geographic units.

3. Table 1 might be more informative by adding the annual values of these labor market variables instead of only showing the changes.

4. To help readers better understand the estimation results, the author might want to add more interpretation of the main estimation results, not just the sign of estimated coefficients but also their magnitudes.

5. Table 3 can be split into two tables with each table testing one hypothesis.

## References

D. Acemoglu and D. Autor. Skills, tasks and technologies: Implications for tmployment and earnings. In *Handbook of Labor Economics*, volume 4, pages 1043–1171. Elsevier, 2011.

D. R. Davis and J. I. Dingel. A spatial knowledge economy. *American Economic Review*, 109(1): 153–70, 2019.

M. Goos, A. Manning, and A. Salomons. Explaining job polarization: Routine-biased technological change and offshoring. *American Economic Review*, 104(8):2509–26, 2014.

T. Taylor, B. Turgeon, A. Buck, K. Bloch, and J. Church. Spatial variation in us labor markets and workplace gender segregation: 1980–2005. *Sociological Inquiry*, 2019.
