# Supplementary material for: Occupational gender segregation and economic growth in U.S. local labor markets, 1980 through 2010
Source: PLoS One. 2020 Jan 14;15(1):e0227615. doi: 10.1371/journal.pone.0227615 (PMC6959984; doi:10.1371/journal.pone.0227615)
Supplement: Attachment — Submitted filename: cover letter to editor_second round.pdf [file pone.0227615.s007.pdf]

Xi Yang
Department of Economics
University of North Texas
Wooten Hall 347
Denton, Texas, 76203
Email: xi.yang@unt.edu

November 25, 2019

Dear Dr. Shihe Fu,

Thank you for the opportunity to review the paper for the PLOS ONE. Attached please see the second round referee report for the paper "Gender segregation of managerial professional occupations and economic growth in U.S. labor markets, 1980 through 2010" (PONE-D-19-24965), which now entitled "Occupational gender segregation and economic growth in U.S. local labor markets, 1980 through 2010".

I enjoyed reading the new draft which I think has a lot of improvements. First, the message in the paper is more focused by using hourly wage as the primary indicator of economic productivity instead of using three different measurements for economic productivity. Second, the estimation strategy is much clear with the additional discussion on the fixed effect model. The concern of reverse causation is explicitly discussed in the new draft, and the new results using the retail trade sector as a counterfactual has helped alleviate this concern. Third, the author added more detailed explanations of the geographic unit of analysis and the definition of occupational gender segregation. Overall, I am satisfied with the answers to the other concerns I raised in my previous report, though there are still some minor points I would like to stress. Most of these comments are clarify questions on the interpretation of some of the new results. They are summarized in the attached report.

Thank you again for this opportunity!

Best wishes,

Xi Yang
