# Supplementary material for: Occupational gender segregation and economic growth in U.S. local labor markets, 1980 through 2010
Source: PLoS One. 2020 Jan 14;15(1):e0227615. doi: 10.1371/journal.pone.0227615 (PMC6959984; doi:10.1371/journal.pone.0227615)
Supplement: Attachment — Submitted filename: referee report_second round.pdf [file pone.0227615.s008.pdf]

Referee Report (second round) for PONE-D-19-24965 Gender segregation of managerial/professional occupations and economic growth in U.S. labor markets, 1980 through 2010" (William Scarborough, 2019)

Summary

I enjoyed reading the new draft. First, the message in the paper is more focused by using hourly wage as the primary indicator of economic productivity instead of using three different measurements for economic productivity.

Second, the estimation strategy is much clear with the additional discussion on the fixed effect model. The concern of reverse causation is explicitly discussed in the new draft, and the new results using the retail trade sector as a counterfactual has helped alleviate this concern.

Third, the author added more detailed explanations of the geographic unit of analysis and the definition of occupational gender segregation, which is helpful for the readers to understand the analytic approach in the paper. I like the new figure on the geographic distribution of occupational segregation which provides readers a straightforward description of occupational segregation.

Overall, I am satisfied with the answers to the other concerns I raised in my previous report. However, there are still some minor points I would like to stress.

1. It is interesting to see that the overall employment effect as presented in the new Table 2 column 1. However, I do not quite understand why the pooled effect is stronger than the effect for FIRE and tech sectors. If the main effect of occupational segregation is driving by the FIRE and tech sectors, as claimed by the author, the size of the estimated coefficient should be smaller for the pooled sample than for the FIRE or tech sectors, right? I think the author might want to provide some discussion of the results here.

2. The new results on economic productivity are presented in table 4, where the hourly wage is used as the measure of productivity. The author found the negative wage effect is stronger among the FIRE industry, but this effect is not significant for the Tech industry. I think the author might want to provide more discussion of why we see different results in the two industries.

3. It would interesting to see the overall wage effects as presented in Table 2 column 1.

4. In the statement of the three hypotheses would be more clear if the author specifies that the occupational gender segregation and sector expansion is measured at local labor market level. For example, hypothesis 1 can be stated as "Lower occupational gender segregation will be associated with expanding finance and tech sectors *at the local labor market level."*
