# Supplementary material for: Occupational gender segregation and economic growth in U.S. local labor markets, 1980 through 2010
Source: PLoS One. 2020 Jan 14;15(1):e0227615. doi: 10.1371/journal.pone.0227615 (PMC6959984; doi:10.1371/journal.pone.0227615)
Supplement: Attachment — Submitted filename: Response to Reviewers.docx [file pone.0227615.s006.docx]

Response to Editor’s and Reviewers’ Comments:

I would like to thank the three reviewers and the editor for their encouragement about the paper and also for their constructive comments. I have revised the paper to address each comment and describe these revisions in detail below. I believe the paper is much improved after incorporating feedback, and I would be happy to make additional revisions that you believe would further improve the paper.

**Similar Comments from Editor and Reviewers:**

1. Both the editor and Reviewer 1 suggested that I specify my focus on “local” labor markets, particularly in the title of the manuscript.

**Response**: I have changed the title of the manuscript to reflect my focus on local labor markets. I have also made changes throughout the manuscript to specify that I’m focusing on local geographic labor markets.

2. The editor, Reviewer 1, and Reviewer 2 each commented on the restricted use of managerial/professional occupational segregation in the original manuscript. Both the editor and Reviewer 1 suggested that I use occupational gender segregation across all occupations. Relatedly, both Reviewer 1 and Reviewer 2 suggested that I add more detail to the measurement of occupational gender segregation.

I have changed my focal predictor to reflect occupational gender segregation across all occupations (as opposed to just managers/professionals) for each industry-labor market-year. These changes are reflected throughout the manuscript, and are described in detail on pages 12 and 13 where I discuss the measurement of occupational gender segregation.

Following Reviewer 1 and 2’s suggestion, I have also added more detail to the measurement of occupational gender segregation. As discussed in the text (pages 12 and 13), I use one of the most common measures of segregation, the index of dissimilarity. Equation 2 (page 12) provides detail on how the index is calculated, and I discuss its interpretation also on page 13.

3. The editor and all three reviewers offered suggestions on additional control variables, including women’s labor force participation, percent with a college degree, percent foreign born, racial segregation, and degree of international trade.

Thank you for the suggestions for additional controls. I have added all recommended covariates. These are discussed in the analytic strategy section on page 13 and reflected in the results in Tables 2 and 3.

4. Reviewers 1 and 3 suggested that I report labor market characteristics for each year in the descriptive section of the results instead of focusing on change.

I have replaced Table 1 with a new table reporting descriptive statistics for each year included in the study.

5. Reviewers 1 and 3 suggested that I discuss the magnitude of coefficients in the results and discuss their economic significance.

Throughout the results section, I have added more interpretation to regression coefficients in order to highlight their economic significance. For example, I now discuss how a change in gender segregation from highly segregated to moderately segregated predicts change in hourly wages. These revisions are present on pages 19 and 23-25.

6. Reviewers 2 and 3 suggested that I specify how standard errors are calculated, with both suggesting that standard errors be clustered by labor markets.

Standard errors are clustered by labor markets. I now report this on page 14 and in Tables 2 and 3.

7. The editor and Reviewer 1 suggested additional citations to studies on urban cultural diversity and productivity.

Thank you for pointing me to the additional citations. They fit well with the framing of the paper around the merits of local diversity. I have added them in the front end of the paper, most noticeably on pages 5 and 6.

**Additional Comments from the Editor**

1. The editor suggested I add a few sentences in the introduction to state my estimation strategy.

I have added text to page 4 stating my use of fixed effects regression models to estimate the relationship of occupational gender segregation to economic outcomes. Following the suggestion of Reviewer 1, (discussed below), I also state my use of retail trade as a counterfactual industry where the hypothesized effects of occupational gender segregation should not be observed as they are in the high-skilled service industries of finance and tech.

2. The editor suggested that I include a pooled model in Table 2 that includes all industries with fixed effects for industry to identify the general effects of occupational gender segregation.

I have added a pooled model to Table 2, including fixed effects for industry. As the editor suggested, this provides insight on the general effects of gender segregation across industries. These changes are reflected in Table 2, on page 19, as well as in my discussion of the estimation strategy on pages 10-12.

3. The editor suggested I explicitly write the econometric models estimated, followed by variable definition.

I have rewritten the analytic strategy section in accordance with the editor’s recommendation. The econometric fixed effects equations are now written explicitly, with corresponding variable definitions following. These changes are reflected on pages 11 through 14.

4. The editor noted an additional limitation to the study: That it cannot identify whether the effects of gender segregation are due to growth through the sharing of information among workers that boosts productivity, or through growth occurring when highly skilled women workers move to labor markets with lower levels of occupational segregation. The editor advised me to acknowledge this limitation, since it is unlikely to be resolved given the current data.

As the editor predicted, I am unable to identify the role of spatial sorting in my results, given the present data. Therefore, I now state this as a limitation on page 30.

5. The editor provided references to studies that have used instrumental variables to study racial segregation as suggestions of how a similar approach may be used to study occupational segregation.

Thank you for this useful reference. Ananat’s use of railroad tracks was a very creative application of instrumental variables to study racial segregation. In my revised manuscript, I have noted my attempts to identify a suitable instrument (pages 11 and 29). Ultimately, I was unable to identify an instrument. However, the suggestion from Reviewer 1, to use a low-skill service industry as a counterfactual (discussed below), helped bolster the claims I make in the paper by showing how the benefits of occupational gender integration to economic growth are experienced primarily in high-skill service industries (finance and tech) where information exchange is more important, while being non-existent in low-skilled service industries (retail trade) where information exchange plays a smaller role.

**Additional Comments from Reviewer 1**

1. Reviewer 1 suggested that I add a map indicating levels of occupational gender segregation across local labor markets to add further descriptive detail to this variable.

I have added Figure 2 to the text, illustrating levels of occupational gender segregation in finance, tech, and retail trade industries across U.S. local labor markets in 2010. I discuss the geographic variation of occupational gender segregation on pages 16 and 17.

2. Reviewer 1 expressed surprise that FIRE increased only by .6 percentage points between 1980 and 2010, when a BLS report showed an increase of 7% between 1979 and 2001.

Thank you for providing the link to the BLS report. The 7% figure referenced from the report refers to FIRE growth as a proportion of its 1979 size (i.e. ∆FIRE from 1979 to 2001/1979 FIRE size), whereas change indicated in Table 1 refers to the percentage point difference in the size of the FIRE sector (i.e. 2010 FIRE – 1980 FIRE). When translating the information in Table 1 to percentage growth (∆FIRE from 1980 to 2010/1980 FIRE size), there is an 11% increase in the size of FIRE industries across labor markets, which is consistent with the 7% increase Reviewer 1 cited as occurring between 1979 and 2001. Additionally, further reading in the BLS report referenced by Reviewer 1 (<https://www.bls.gov/opub/mlr/2016/article/current-employment-statistics-survey-100-years-of-employment-hours-and-earnings.htm>) indicates that the size of the FIRE sector was 4.6 percent of employment in 1949 and 5.7 percent of employment in 2015 – consistent with the figures reported in Table 1.

3. Reviewer 1 pointed out the potential for reverse causation between occupational gender segregation and economic outcomes. The reviewer suggested that, if unable to identify a valid instrument, I acknowledge this limitation. The reviewer also suggested that I examine a low-skill industry where the sharing of information would be less related to economic growth as a counterfactual to the high-skill industries of finance and tech.

Thank you for these helpful suggestions to my methods. As suggested, I have noted the potential for endogeneity related to reverse causality (pages 11 and 29). I have also followed Reviewer 1’s suggestion to use a low-skill service industry as a counterfactual case. I use retail trade as a counterfactual. I believe this suggestion greatly improved the validity of my findings because the counterfactual industry would be similarly affected by omitted variables as finance and tech, but would not be similarly affected by the mechanism of interest – how occupational gender segregation facilitates information exchange to improve economic output.

I discuss the use of a counterfactual on pages 4, 11, and throughout the results section. As expected if occupational segregation operates through the hypothesized mechanism, no significant effects were observed in retail trade, while they were present in finance and tech.

4. Reviewer 1 suggested that I focus on wages as a primary indicator of economic productivity.

Following Reviewer 1’s suggestion, I now focus primarily on hourly wages as an indicator of economic productivity in finance, tech, and retail trade sectors.

5. Reviewer 1 recommended that I discuss the difference between labor sheds and metros / commuting zones.

I have added text to page 9 to specify how labor sheds offer an advantage over metro areas in delineating commuting zones where individuals live and work.

6. Reviewer 1 suggested I split Table 3 into two tables corresponding to hypotheses 2 and 3.

In this revision, I did not generate an additional table because I reduced my focus from three economic outcomes (wages, household income, establishment growth) to wages as an indicator of productivity (following Reviewer 1’s suggestion from above). By pairing direct and moderating effects in Table 3, I am able to succinctly interpret the full association of occupational gender segregation to wages for each industry. If the reviewer continues to feel that splitting Table 3 into two tables would be ideal, I would be very willing to do so.

**Additional Comments from Reviewer 2**

1. Reviewer 2 recommended further justification for the use of labor sheds as local labor markets.

On page 9, I have added further justification for the use of labor sheds. In particular, I state that labor sheds are the best measure for commuting zones. Because the hypothesized effects of occupational gender segregation on economic outcomes occurs through exchange between workers, labor sheds are an ideal unit of analysis by virtue of delineating the boundaries where employees work and reside. Further following the reviewer’s suggestion, I also focus on specific industries within labor markets (pages 11-13) to engage more precisely with the mechanisms by which occupational gender segregation affects economic outcomes.

2. Reviewer 2 suggested that I discuss limitations related to omitted variable bias.

As the reviewer suggested, my use of fixed effects models is unable to control for unobserved time variant characteristics. I state this limitation on pages 11 and 29.

**Additional Comments from Reviewer 3**

1. Reviewer 3 suggested examining other levels of the wage distribution besides the median to test whether results remain consistent.

I have added Appendix B to the manuscript reporting results of models predicting wages at the 25^th^, 50^th^, and 75^th^ percentile. Findings are generally consistent with those reported in the main text focusing on the median. However, effects are strongest at the 75^th^ percentile. This is consistent with the fact that this part of the wage distribution corresponds to high-skill workers most likely to benefit from the exchange of information occurring in low-gender segregated environments.

Because the findings were consistent across the wage distribution, and because the study focuses on the general relationship of occupational gender segregation to economic productivity, rather than the wage distribution within industries, I discuss median wages in the main text and report other aspects of the wage distribution in Appendix B.

2. Reviewer 3 recommended that the r-squared for each model be added to Tables 2 and 3.

I have added the r-squared to each model in Tables 2 and 3. Thank you for your attention to detail.

3. Reviewer 3 suggested that I visualize interactions by illustrating how the effect of industry growth changes depending on levels of occupational gender segregation.

I have added Figure 3 which reports the predicted coefficient for industry growth across varying levels of occupational segregation.
