# Supplementary material for: Occupational gender segregation and economic growth in U.S. local labor markets, 1980 through 2010
Source: PLoS One. 2020 Jan 14;15(1):e0227615. doi: 10.1371/journal.pone.0227615 (PMC6959984; doi:10.1371/journal.pone.0227615)
Supplement: Attachment — Submitted filename: Letter to Reviewers2.docx [file pone.0227615.s009.docx]

Response to Editor and Reviewer Comments:

Thank you for the opportunity to revise my manuscript and for the valuable comments. I have followed all suggestions, and believe the article has further improved. These changes are summarized below.

**Comments from Editor**

1. The editor suggested I correct a statement on page 11 referring to the challenge of identifying a suitable instrumental variable.

**Response**: Thank you for identifying the problem with this statement. I have followed the suggestion and stated, as suggested, that I was unable to find an appropriate instrumental variable in the case of this study.

**Comments from Reviewer:**

1. The reviewer noted that the coefficient in the pooled model in Table 2 for the effect of occupational gender segregation was not intuitive, since it was much larger in magnitude than what was observed in subsequent equations independently modelling the effect by industry. The reviewer suggested that I clarify why this pattern is observed.

**Response**: I have added text to page 21 noting the larger coefficient in the pooled model compared to the models for FIRE and tech and providing an explanation. While the pooled model controls for unobserved characteristics of industries through a fixed effect term, it does not account for the varying effects of controls across industries. The subsequent equations run separately by industry allow for this level of detail. For example, the size of export-intensive industries has no effect on FIRE growth, a positive effect on Tech growth, and a negative relationship with growth in retail trade. Controlling for these types of varying effects of controls mediated the coefficient for occupational segregation from what would be expected given its size in the pooled model. Nonetheless, the coefficients remain significant and substantial.

2. The reviewer suggested that I provide more discussion of why the direct effects of occupational gender segregation on wage growth were significant for FIRE but not for tech.

I have added text to pages 27 and 28 discussing why there were differing direct effects of occupational gender segregation on wages in FIRE and tech industries. While both FIRE and tech industries grew between 1980 and 2010, tech grew at over twice the rate as FIRE. In the context of such large growth, industry expansion played a more prominent role in wages in tech. This is consistent with the fact that the coefficient for industry expansion on wages is over twice as large for tech as FIRE. Furthermore, the results illustrated in Figure 3b show that the effect of occupational gender segregation on wages in tech occurs primarily in the context of industry expansion (noted on page 28).

3. The reviewer suggested that I include models reporting results for the pooled sample in Table 3, mirroring the approach done in Table 2.

I have added two additional columns to Table 3 reporting results for a pooled model including all industries and with an added industry fixed effect. Discussion of these results has been added to page 24, where I also note the limitations of the pooled model, which is unable to examine varying effects of predictors across industries. This provides a suitable set-up for subsequent models that more directly test the industry-specific hypotheses using independent equations that control for the varying effects of control variables and focus on the effects of occupational gender segregation in FIRE, tech, and retail trade.

4. The reviewer recommended that I clarify the hypotheses to specify measurement at the local labor market level.

Thank you for the specific suggestion of language that would clarify the hypotheses. I have directly incorporated this recommendation to each hypothesis, located on pages 8 and 9.
