# Supplementary material for: Occupational gender segregation and economic growth in U.S. local labor markets, 1980 through 2010
Source: PLoS One. 2020 Jan 14;15(1):e0227615. doi: 10.1371/journal.pone.0227615 (PMC6959984; doi:10.1371/journal.pone.0227615)
Supplement: Attachment — Submitted filename: Referee Report.docx [file pone.0227615.s005.docx]

Referee Report

Title: Gender segregation of managerial/professional occupations and economic growth in U.S. labor markets, 1980 through 2010

This paper uses 1980, 1990, and 2000 census data with the 2010 American Community Survey 5-year microdata sample to form a panel data at the labor-shed level to empirically study the relationship between labor market levels of gender segregation among managerial/professional occupations and economic growth as well as the effect on labor market economic well-being (median hourly wages, median household income, and growth in the number of business establishments). The results show that a lower level of gender segregation helps expand in the finance and the tech sectors. And, in general, a lower level of gender segregation increases median hourly wages, median household income, and business growth.

This paper is interesting and has important policy implications. The paper is well-written. I recommend accepting this paper under the condition that the following issues are addressed.

1. Does the sample used in the regression analysis include immigrants? In the US, immigrant ratios in local labor markets could be possibly correlated with gender segregation and determine economic growth and economic well-being, resulting in biased and inconsistent results. The two-way fixed effects model may not address this issue.
2. Table 1 reports the change in labor market characteristics, not the level. I suggest addionally report the level of the key variables can offer better information about the data used.
3. Why use median hourly wages and median household income, not the mean? The paper does not give a reason. Is it because large outliers in the two variables so the paper uses medians? If using medians for the dependent variables, why not doing quantile regressions and show other interesting results such as quartiles or deciles?
4. The paper did not discuss the economic significance of the estimated coefficients. That is, for example on page 17, do the sizes of the estimates make sense? At least some discussion will be helpful.
5. What kind of standard error does the paper use? I did not find it. The tables simply report “Standard errors in parentheses”. Since the paper uses panel data regressions, reporting clustered standard errors helps reduce the efficiency concern.
6. Tables 2 and 3 should report R^2^.
7. Models in Table 3 use interaction terms and report all estimated coefficients, which is hard to interpret the results regarding the effect of gender segregation on each of the outcome variables. I suggest to additionally report marginal effects in the table. A better way is to draw a plot to show how the effect changes at different levels of gender segregation.
